# RETRACTION: Effect of Open Versus Minimally Invasive Surgery on Postoperative Wound Site Complications in Patients With Recurrent Shoulder Instability: A Meta‐Analysis

**DOI:** 10.1111/iwj.70353

**Published:** 2025-03-17

## Abstract

**RETRACTION:** PanD. , SuoY. , ChenQ. , HouD. , and ZhangL. , “Effect of Open Versus Minimally Invasive Surgery on Postoperative Wound Site Complications in Patients With Recurrent Shoulder Instability: A Meta‐Analysis,” International Wound Journal 21, no. 2 (2024): e14412, 10.1111/iwj.14412.PMC1082461737751908

The above article, published online on 26 September 2023, in Wiley Online Library (http://onlinelibrary.wiley.com/), has been retracted by agreement between the journal Editor in Chief, Professor Keith Harding; and John Wiley & Sons Ltd. Following an investigation by the publisher, all parties have concluded that this article was accepted solely on the basis of a compromised peer review process. The editors have therefore decided to retract the article. The authors did not respond to our notice regarding the retraction.

**RETRACTION:** D. Pan , Y. Suo , Q. Chen , D. Hou , and L. Zhang , “Effect of Open Versus Minimally Invasive Surgery on Postoperative Wound Site Complications in Patients With Recurrent Shoulder Instability: A Meta‐Analysis,” International Wound Journal 21, no. 2 (2024): e14412, 10.1111/iwj.14412.PMC1082461737751908

The above article, published online on 26 September 2023, in Wiley Online Library (http://onlinelibrary.wiley.com/), has been retracted by agreement between the journal Editor in Chief, Professor Keith Harding; and John Wiley & Sons Ltd. Following an investigation by the publisher, all parties have concluded that this article was accepted solely on the basis of a compromised peer review process. The editors have therefore decided to retract the article. The authors did not respond to our notice regarding the retraction.

